# Metallothionein Induction in the Coelomic Fluid of the Earthworm *Lumbricus terrestris* following Heavy Metal Exposure: A Short Report

**DOI:** 10.1155/2014/109386

**Published:** 2014-04-03

**Authors:** A. Calisi, M. G. Lionetto, E. De Lorenzis, A. Leomanni, T. Schettino

**Affiliations:** Department of Biological and Environmental Sciences and Technologies (DiSTeBA), University of Salento, Via Provinciale Lecce-Monteroni, Monteroni, 73100 Lecce, Italy

## Abstract

Earthworms are useful bioindicator organisms for soil biomonitoring. Recently the use of pollution biomarkers in earthworms has been increasingly investigated for soil monitoring and assessment. Earthworm coelomic fluid is particularly interesting from a toxicological perspective, because it is responsible for pollutant disposition and tissue distribution to the whole organism. The aim of the present work was to study the effect of heavy metal exposure on metallothionein (Mt) induction in the coelomic fluid of *Lumbricus terrestris* in view of future use as sensitive biomarker suitable for application to metal polluted soil monitoring and assessment. *L. terrestris* coelomic fluid showed a detectable Mt concentration of about 4.0 ± 0.6 **μ**g/mL (mean ± SEM, *n* = 10) in basal physiological condition. When the animals were exposed to CuSO_4_ or CdCl_2_ or to a mixture of the two metals in OECD soils for 72 h, the Mt specific concentration significantly (*P* < 0.001) increased. The Mt response in the coelomic fluid perfectly reflected the commonly used Mt response in the whole organism when the two responses were compared on the same specimens. These findings indicate the suitability of Mt determination in *L. terrestris* coelomic fluid as a sensitive biomarker for application to metal polluted soil monitoring and assessment.

## 1. Introduction

Soil pollution has enormously increased during the last decades due to industrial activities, urban waste, intensive use of biocides and fertilizers, and atmospheric deposition. The most diffusive pollutants occurring in soil are heavy metals, which can enter the soil from different sources, such as pesticides, fertilizers, organic and inorganic amendments, mining, wastes, and sludge residues [1]. In contrast to harmful organic compounds, heavy metals do not decompose and do not disappear from soil even if their release to the environment can be restricted [2]. Therefore, the effects of heavy metal contamination on soil organisms and decomposition processes persist for many years.

Earthworms are very important organisms for soil formation and organic matter breakdown in most terrestrial environments and they have been considered to be convenient indicators of land use impact and soil fertility [3]. Because of their particular interactions with soil, they are significantly affected by pollutants reaching the soil system. On the other hand, they can influence pollutant availability in the soil with their burrowing activity [4]. Earthworms can be exposed to contaminants in two principal ways. Firstly, living in the soil they are in direct contact with soil pore water and therefore with pollutants therein dissolved. The earthworm skin is extremely permeable to water [5] and it represents a main route for contaminant uptake [6, 7]. Secondly, these organisms ingest large amounts of soil; therefore, they are continuously exposed to contaminants adsorbed to solid particles through their alimentary tract [8]. Previous studies under both field and laboratory conditions have demonstrated that earthworms bioaccumulate certain metals (such as Cd, Cu, Zn, Pb, and Mo) from soil [9–12]. They tolerate high tissue metal concentrations [12–14] due to well-developed trafficking and storage pathways for heavy metals, including metallothioneins (Mt) [15]. Mts are low-molecular-weight cysteine-rich metal-binding proteins that are involved in regulation of metabolism of trace metals and protection against heavy metal toxicity and oxidative stress [16, 17] in a wide range of phylogenetic groups [18–20], including earthworms [21–23]. Earthworms express two distinct MT isoforms (MT-1 and MT-2) characterized by a high cysteine content and no significant aromatic residues [24]. Their metal-responsiveness was confirmed by determining Mt-specific expression profiles in earthworms exposed to soils of differing heavy metal concentrations. The two isoforms have different subcellular distributions and functions, namely, the homeostasis and detoxification of essential and nonessential metals, respectively [13, 24, 25].

Although the amino acid sequences of more than 50 invertebrate MT and MT-like proteins have already been determined, little is known about the biochemical properties of earthworm MTs. So far, only 5 MT genes of earthworms have been cloned from* Lumbricus castaneus*,* Eisenia fetida*,* Lumbricus rubellus*, and* Lumbricus terrestris* [26, 27] whose expression is differentially regulated by different heavy metals [13, 24, 28]. The utility of Mt induction in earthworms as reliable biomarker in monitoring and assessment soil metal pollution has been demonstrated by several studies on different earthworm species [28–30].

Previous studies demonstrated a significant induction of Mt proteins in different earthworm species such as* Lumbricus rubellus*,* Eisenia fetida*, and* Eisenia andrei* exposed to cadmium [31–33] or in* Lumbricus mauritii* exposed to Pb and Zn contaminated soil [27] and in* Lumbricus terrestris* exposed to cadmium, copper, and mercury [30, 34, 35]. Morgan et al. [8] previously described the immunoperoxidase localization of Mt in the major organs and tissues of the earthworm* Lumbricus rubellus* sampled from a mine soil heavily polluted with Pb, Zn, and Cd. MT expression was strongly detected in the chloragogenous tissue and also in the apical cytoplasm of intestinal epithelial cells, within the narrow tubular region of nephridia, in the secretory epithelia of the calciferous gland, and in coelomocytes contiguous with chloragocytes attached to the gut. Homa et al. [36, 37] described Mt gene induction in coelomocytes of the earthworms* Eisenia fetida* and* Allolobophora chlorotica* after three days of dermal exposure to metals.

Earthworm coelomic fluid is particularly interesting from a toxicological perspective, because it is responsible for pollutant disposition and tissue distribution to the whole organism. Its cells (coelomocytes) are involved in the internal defense system [38–40] and any impairment of coelomocyte functioning can compromise the health of the entire organism. Therefore, this physiological fluid is very interesting for the development of novel nondestructive pollution biomarkers.

The aim of the present work was to study the effect of metal exposure on Mt induction in the coelomic fluid of the earthworm* L. terrestris* in view of future use as sensitive nondestructive biomarker suitable for metal polluted soil monitoring and assessment.

We selected* L. terrestris* because of its ecological habits in the soil. It is an anecic species that forms temporary deep burrows and comes to the surface to feed [41]. Therefore, it can be exposed to pollutants present not only in the soil surface but also in the soil deeper layer. It has a large size which permits handling and collection of enough amounts of coelomic fluid without compromising the survival of the animal.* L. terrestris* specimens were exposed to copper sulphate or cadmium chloride or a mixture of the two metals in OECD soil as described in the Section 2.

## 2. Methods

### 2.1. Experimental Design

A homogeneous group of earthworms (*L. terrestris*) (*n* = 130) of the same size (body weight of depurated—void gut—earthworms was 2.14 ± 0.18 g) was acclimated for 48 h in 11 boxes (10 animals per box) each containing 1 kg of soil at 18 ± 1°C and 16 : 8 h light/dark regime and located in a climatized room.

The OECD soil utilized was a mixture of 10% sphagnum peat, 20% kaolin clay, and 70% air-dried sand with more than 50% of the particles between 50 and 200 microns according to the OECD guidelines [42]. The dry constituents were blended in the correct proportions and mixed thoroughly. The soil moisture content was adjusted to 45% of the water holding capacity with deionized water. The pH was 5.6 adjusted to 6.0 with calcium carbonate [43]. The constructed soil represented the drilosphere soil.

After this acclimation period 20 specimens were utilized for the determination of Mt concentration in the coelomic fluid and in the whole organism in basal physiological conditions. The remaining 110 specimens were utilized for the exposure experiment. They were divided into five subgroups: the first (*n* = 30) was utilized as initial control (at time 0), the second (*n* = 20) was exposed to copper sulphate, the third (*n* = 20) was exposed to cadmium chloride, the fourth (*n* = 20) was exposed to a mixture of the two metals, and the fifth was utilized as final control to be analyzed at the end of the exposure experiment. The exposure time was 72 h. As previously demonstrated [35], this time is sufficient to induce a significant response to heavy metal exposure in* L. terrestris*. The toxicant concentrations utilized were 45 mg/kg of soil for copper sulphate and 1 mg/kg of soil for cadmium chloride, respectively. They are far below the EC50 values reported for copper and cadmium in earthworms [44]. These concentrations represent copper sulphate and cadmium chloride intervention values, which are generic soil quality standards based on potential risks to human health and ecosystems as indicated in DM number 471 25 October 1999 [45]. The toxicants were dissolved in water and added to the terrain at the start of the exposure experiment. The exposure to the mixture of the two metals was performed by adding each metal to the soil at the same concentration used for the single exposure.

At time 0 and 72 hours coelomic fluid was sampled from each specimen by puncturing postclitellum segments of the coelomic cavity by a sterilized hypodermic syringe (1 mL syringe, needle 27G). Coelomic fluid sampling did not compromise the animal survival.

### 2.2. Mt Measurement

Mt coelomic concentration was determined by the spectrophotometric method previously described [46, 47] in either the whole organism or the coelomic fluid. Briefly, tissues were homogenized (1 : 3 wt/vol for whole organism or 1 : 3 vol/vol for coelomic fluid) in the following buffer: 0.5 M sucrose, 20 mM Tris-HCl buffer, pH 8.6, added with 0.006 mM leupeptin, 0.5 mM phenylmethylsulphonilfluoride as antiproteolytic agents, and 0.01% *β*-mercaptoethanol as a reducing agent. Then, the homogenate was treated to obtain a partially purified Mt fraction by ethanol/chloroform precipitation. Mt concentration in the samples was quantified by spectrophotometric titration of the sulphydryl residues using the Ellman's reagent (5,5′-dithiobis-2-nitrobenzoic acid) using reduced glutathione (GSH) as a standard. Data were expressed as *μ*g MT/mg of proteins.

Protein concentration was quantified by the Bradford assay [48] using NanoDrop ND-1000 UV-Vis (Thermo Scientific, Waltham, MA, U.S.A.).

### 2.3. Electrophoresis

Coelomic fluid homogenate (see above) of control and heavy metal exposed animals following 72 hours of exposure was analyzed by sodium-dodecyl-sulfate polyacrylamide gel electrophoresis on 1 mm thick slab gels according to the method of Laemmli [49] with reducing conditions (5% (V/V) 2-mercaptoethanol) using Protean II Xi cell (Bio-Rad Laboratories, Marnes La Coquette, France) and acrylamide/bisacrylamide slab gels (15% T, 2.67% C, 375 mM Tris-HCl, pH 8.8 containing 0.1% (W/V) SDS). Pure rabbit Mt (SIGMA, St. Louis, USA) was analyzed in parallel. The separation was performed at 15°C using Laemmli buffer (25 mM Tris, 192 mM glycine, and 0.1% (W/V) SDS, pH 8.3 from Bio-Rad) for 5 min at 200 V, then for 3.5 h at 350 V. The gel was stained with Coomassie Brilliant Blue for protein detection.

### 2.4. Statistical Analysis

Data were analyzed by two-way ANOVA and Newman Kewls posttest using a two factor orthogonal experimental design: factor (A) “metal exposure” which included four levels (“not exposed” or control animals, “Cd exposed,” “Cu exposed,” and “Cd + Cu exposed”) and factor (B) “tissue” which included two levels (“coelomic fluid” and “whole organism”). WinGmav 5 software (designed, coded, and compiled by A. J. Underwood and M.G. Chapman, Institute of Marine Ecology, University of Sydney, Australia) was used. The homogeneity of variance was previously tested by Cochran's test. Data are expressed as mean ± S.E.M.

## 3. Results

*L. terrestris* coelomic fluid showed a detectable Mt concentration of about 4.0 ± 0.6 *μ*g/mL (mean ± SE, *n* = 10) in basal physiological condition.

**Figure 1:**
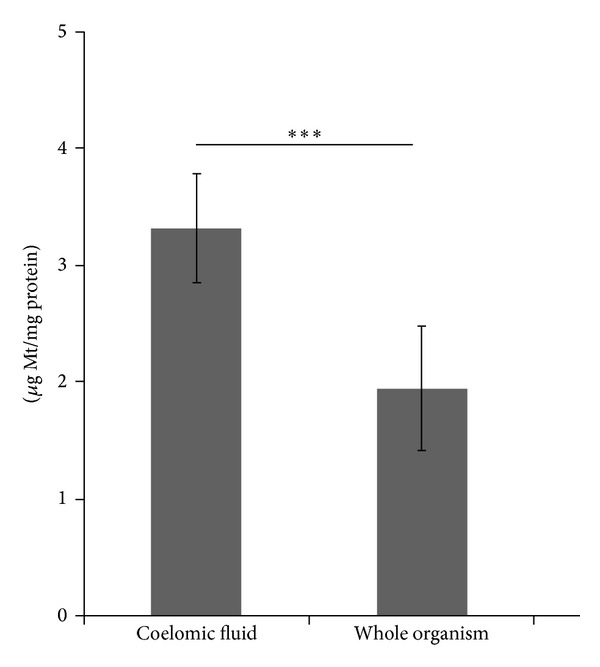
Mt specific concentration (expressed as *μ*g·mg^−1^ proteins) in the coelomic fluid and in the whole organism determined in unexposed* L. terrestris*. ***P < 0.001 (student's* t*-test).

With the aim to compare the MT concentration in the coelomic fluid with the Mt concentration determined in the whole organism, the specific MT concentration, referred to the protein content of the sample, was calculated. As presented in Figure 1, the Mt specific concentration in the coelomic fluid appeared significantly higher with respect to the MT concentration determined in the whole organism.

**Figure 2:**
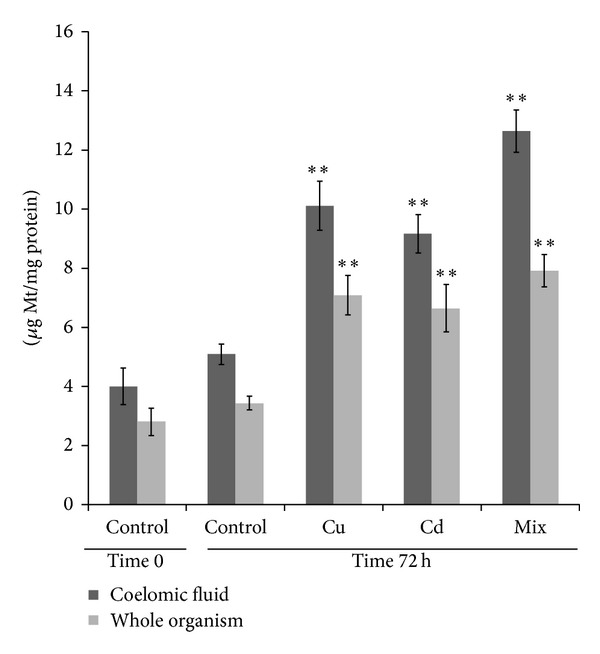
Mt specific concentration (expressed as *μ*g·mg^−1^ proteins) in the coelomic fluid and in the whole organism determined in control and metal exposed* L. terrestris* (see Section 2). The statistical significance of data was analyzed by two-way ANOVA and Newman Kewls posttest (see Section 2). **P < 0.01.

**Figure 3:**
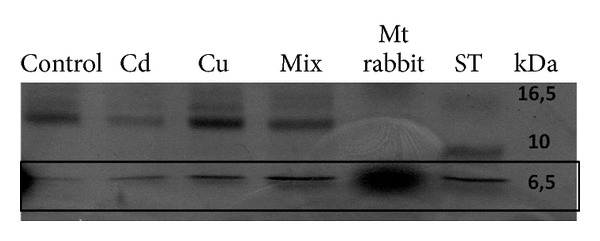
SDS gel electrophoresis of* L. terrestris* coelomic fluid. Line 1: coelomic fluid from control specimens; line 2: coelomic fluid from copper sulphate exposed specimens; line 3: coelomic fluid from cadmium chloride exposed specimens; line 4: coelomic fluid from specimens exposed to a mixture of the two metals; line 5: pure rabbit Mt; line 6: molecular mass markers.

When the animals were exposed for 72 h to CuSO_4_ or CdCl_2_ or to a mixture of the two metals in OECD soils, the Mt specific concentration significantly (*P* < 0.001) increased both in the coelomic fluid or in the whole organism with respect to control group, as indicated in Figure 2. On the other hand, control animals did not show any significant changes with respect to time 0 following 72 h of exposure under the same conditions of test organisms except for the absence of metals in the soil. In all the cases, the Mt specific concentration was significantly (*P* < 0.001) higher in the coelomic fluid with respect to the whole organism. As demonstrated by the two-way ANOVA, the two variability factors “metal exposure” and “tissue” (see Section 2), exerted an extremely significant effect, accounting for 44.17% and 20.37% of the total variance, respectively. There was no significant interaction between the two variability factors, suggesting that metal exposure induced a detectable increase in the Mt expression in the same way on both coelomic fluid and the whole organism. The percentage of Mt induction in the whole organism or coelomic fluid did not vary significantly between the metal exposure conditions (CuSO_4_, CdCl_2,_ and mix) (Table 1). The electrophoretic analysis of the coelomic fluid (Figure 3) confirmed the results obtained spectrophotometrically, showing a significant increase in intensity of the bands corresponding to Mt in samples exposed to heavy metals.

## 4. Discussion

Earthworm biomarkers are becoming increasingly important in the recent years in the evaluation of the effects of contaminants on soil organisms [50–52]. They are considered useful tools in soil monitoring and assessment as an early warning of adverse ecological effects [52, 53]. This has increased the interest in the study of earthworm biological responses to pollutants. Earthworm physiological fluids, such as coelomic fluids and blood, offer an interesting field for exploitation of novel sensitive nondestructive biomarkers. In long-term biomonitoring of native populations inhabiting polluted areas, the use of nondestructive biomarkers as markers of condition becomes of relevance.

In the present study, we assessed the suitability of the Mt determination in the coelomic fluid of the earthwork* L. terrestris* as sensitive biomarker of exposure to heavy metals.

In physiological conditions* L. terrestris* coelomic fluid showed a significant basal concentration of Mt. It is known that earthworm coelomocyte population is comprised of amoebocytes originating from mesenchymal lining of the coelom [54] and eleocytes (chloragocytes) sloughed into the coelomic fluid from the chloragogen tissue surrounding the intestine and blood vessels [55]. As demonstrated by Morgan et al. [8], chloragocytes, as well as the chloragogenous tissue from which they derive, are able to synthetize Mt. Mt concentration detected in the coelomic fluid in the present study probably represented the Mt concentration of the above cells.

When the animals were exposed to heavy metals such as copper sulphate or cadmium chloride in OECD soil for 72 h the coelomic fluid, MT concentration significantly increased, as indicated by both the electrophoretic profile of the coelomic fluid and the spectrophotometric analysis of the coelomic fluid Mt enriched fraction. This result is in agreement with results found by Homa et al. [36, 37] who found Mt gene induction in coelomocytes of the earthworms* Eisenia fetida* and* Allolobophora chlorotica* after dermal exposure to metals for three days. The responses observed in* L. terrestris *following exposure to 45 mg/kg of soil for copper sulphate or 1 mg/kg of soil for cadmium chloride respectively were almost the same, suggesting the higher sensitivity of* L. terrestris* to cadmium with respect to copper.

In order to compare the Mt response in the coelomic fluid with the most utilized destructive measure of the Mt concentration in the whole organism, the two determinations were performed in parallel on the same specimens. The obtained results demonstrated that the Mt response in the coelomic fluid perfectly reflects the Mt response in the whole organisms both qualitatively and quantitatively.

These findings indicate the suitability of Mt determination in* L. terrestris* coelomic fluid as nondestructive biomarker for application to metal polluted soil monitoring and assessment.

The use of coelomic fluid for Mt detection in* Lumbricus terrestris* offers many advantages with respect to the commonly used whole-body measure, particularly for in-field application. In fact, coelomic fluid can be easily sampled in native animals in field without compromising the animal survival, avoiding the stressful translocation to the laboratory. Moreover, considering the recent development of novel biomarkers in the earthworm coelomic fluids [22], the standardization of the Mt determination contributes to the development of multiple assays on this important earthworm tissue.

## Figures and Tables

**Table 1:** Percentage variation of metallothionein concentration in *L. terrestris* coelomic fluid or whole organism following 72 h exposure to CdCl_2_, CuSO_4_ or a mixture of the two metals in OECD soil (for the concentration used see Section 2). The percentage variation was calculated according to the formula: (treated − control/control) × 100.

	Coelomic fluid	Whole organism
CuSO_4_	99.0 ± 6.5	106.4 ± 9.4
CdCl_2_	80.4 ± 9.4	93.8 ± 11.5
CdCl_2_ + CuSO_4_	149.0 ± 6.5	130.5 ± 7.0

Data are reported as mean ± S.E.M.
